# Protocol for a phase I trial of a novel synthetic polymer nerve conduit 'Polynerve' in participants with sensory digital nerve injury (UMANC)

**DOI:** 10.12688/f1000research.19497.1

**Published:** 2019-06-24

**Authors:** Ralph Murphy, Alessandro Faroni, Jason Wong, Adam Reid

**Affiliations:** 1 Blond McIndoe Laboratories, Division of Cell Matrix Biology and Regenerative Medicine, School of Biological Sciences, Faculty of Biology, Medicine and Health, The University of Manchester, Manchester Academic Health Science Centre, Manchester, M13 9PT, UK; 2 Department of Plastic Surgery & Burns, Wythenshawe Hospital, Manchester University NHS Foundation Trust, Manchester Academic Health Science Centre, Manchester, UK

**Keywords:** Peripheral nerve injury, nerve conduit, biomaterials, Poly-ε-caprolactone, Poly-L-lactic acid

## Abstract

**Background:** Peripheral nerve injuries are common, with approximately 9,000 cases in the UK annually. Young working individuals are predominantly affected, leading to significant health and social implications. Functional recovery is often poor with impaired hand sensation, reduced motor function and pain and cold intolerance. Where a nerve gap exists, nerve grafting remains the gold-standard treatment but creates a second surgical site, sensory deficit at the donor site, possible neuroma formation and has limited availability. Current commercially available synthetic and resorbable nerve conduit alternatives are reported to be rigid and inflexible. This study will set out to examine the first-in-man use of a new nerve conduit device ‘Polynerve’ to repair small nerve gaps in digital sensory nerves of the hand. Polynerve is a degradable co-polymer of poly-ε-caprolactone and poly-l-lactic acid, which is shaped as a cylinder that has greater tensile strength, flexibility and less acidic degradation compared with current commercially available synthetic nerve conduits. In addition, it has a novel micro-grooved internal lumen that aids Schwann cell ingress and alignment to improve nerve regeneration.

**Methods: **In total, 17 eligible participants will be recruited to undergo repair of a transected sensory nerve of the hand using the Polynerve device. All participants that receive the nerve conduit device will be followed for a period of 12 months post-surgery. The primary endpoint is safety of the device and the secondary endpoint is degree of sensory nerve regeneration through the conduit assessed using standard sensory testing (2-PD, WEST monofilament testing and locognosia).

**Discussion: **The ‘UMANC’ trial is a single-centre UK-based, prospective, unblinded, phase I clinical trial of a novel nerve conduit device. We aim to demonstrate the safety of Polynerve as a synthetic, biodegradable nerve conduit and improve the treatment options available to patients with significant nerve injuries.

**Registration: **Clinicaltrials.gov: NCT02970864; EudraCT: 2016-001667-37.

## Introduction

Peripheral nerve injuries are a common occurrence, with approximately 9000 cases in the UK occurring each year. Most are in a predominantly young and working population. Where surgical reconstruction is required to repair the peripheral nerve injury, techniques employed have changed little in the last 50–60 years ^1^ with many factors influencing the outcomes, such as age of patient, timing, level and extent of injury, method of repair and the surgeon’s skill ^2^.

Despite advances in microsurgical nerve repair techniques, functional recovery is often poor, and can result in impaired hand sensation, reduced motor function and frequent pain and cold intolerance. This can have a profound and permanent impact on the patient’s recovery and subsequent quality of life. Nerve repair has significant health, social and cost implications with the treatment and rehabilitation of an employed person, estimated to be €51,238 ^3^.

Peripheral nerve injury usually presents with nerve stumps that can be approximated in surgical repair: direct, end-to-end suture repair of the epineurium (neurorrhaphy). Excessive tension over the suture line leads to poor results ^4^; therefore, when the nerve stumps cannot be approximated without tension, an alternative surgical method is required.

Where the nerve gap exceeds more than 5 mm, there are two fundamental options, either ‘nerve grafting’ or ‘tubulisation’ using a bridging material ^5^. This study will examine the first-in-man use of a new nerve conduit device ‘Polynerve’ to repair small nerve gaps in digital sensory nerves of the hand. Polynerve is a degradable co-polymer of poly-ε-caprolactone (PCL) and Poly-L-lactic acid (PLLA) which is shaped as a cylinder with a novel internal lumen consisting of a specific micro-grooved architecture.

### Current treatments

In the presence of a nerve gap, the gold standard is to use the patients’ own nerve tissue (autograft) to bridge the gap in the damaged/severed nerve. The sural/antebrachial cutaneous/posterior interosseus nerves are favourable and commonly used donor nerves to use in such repair. As the harvested nerve undergoes Wallerian degeneration, the autograft functions as a guide or scaffold, with the advantage of the presence of Schwann cells creating a conducive environment for regenerating axons ^1^.

However, there are inherent problems in this approach: a second surgical site and sensory deficit at the donor site, limitation of material, possible neuroma formation and graft mismatch ^5– 8^.

This has prompted research on the use of bio-engineered nerve conduits as an artificial means of guiding axonal regeneration. These conduits function as a guide for axonal growth and help maintain an internal environment for nerve regeneration. Currently available nerve conduits are not ideal: several are prepared from inert non-biodegradable materials or from natural collagen with inherent risks of disease transmission and immuno-rejection. Furthermore, the high cost of these devices limits their appeal to the United Kingdom’s National Health Service, where cost-effectiveness is paramount. Despite advances in bioengineering, commercially available conduits to date have not been able to match the results of the current clinical gold standard, i.e. that of reconstruction using a nerve graft.

### Review of peripheral nerve conduits

Nerve repair by tubulisation, in which the opposing nerve stumps of a transected nerve are enclosed in a tube (nerve guide conduit), was first described in 1880 when a decalcified bone segment was used to provide a pathway between the ends of a severed nerve ^9^. The tubulisation technique is designed to protect the nerve from the surrounding tissue and potential scar tissue, as well as providing mechanical support, directional guidance for axonal sprouting and an environment permissive to nerve development and regeneration ^2, 10^. A number of reviews have been published in which the ideal features of a nerve guide conduit are considered; that is, readily available in appropriate sizes, sterilisable, easily implantable, biocompatible, and with appropriate physical and mechanical characteristics to be flexible and suturable, yet able to withstand compression or collapse ^2, 11^.

### Non-resorbable peripheral nerve guidance conduits

The first generation of artificial nerve guide conduits for clinical application were non-resorbable silicone tubes however in many cases further surgeries were required to remove the tubes following nerve regeneration due to the risk of nerve compression ^6, 12^. Although silicone and expanded polytetrafluroethylene (ePTFE) have both been used clinically for nerve repair, use of non-resorbable materials in this indication has been associated with chronic nerve compression, decreased axonal conduction and fibrosis ^8, 11^ as well as presenting a risk of chronic foreign body reaction with excessive scar tissue formation ^7^.

As a result of the problems experienced, use of non-resorbable materials in nerve guide conduits has significantly declined. Furthermore, in describing the design criteria for nerve guidance conduits the FDA, who have responsibility for regulatory approval of medical devices for use in the USA, have stated that the material used must be biodegradable ^10^. Conduit dimensions are also highlighted as a critical design feature as diameter and wall thickness are believed to influence the rate of regeneration as well as potentially compressing the growing nerve if the diameter is inadequate ^10^.

### Resorbable natural peripheral nerve guidance conduits

Resorbable nerve guidance conduit devices may be derived from natural or synthetic materials. Natural polymer-based devices are predominantly derived from collagen because of the inherent biocompatibility and relative abundance of this protein ^13^. A number of Type I bovine collagen derived nerve guidance conduits and wraps have gained regulatory approval for use in peripheral nerve injury: i.e. NeuraGen® (Integra, FDA 510 (k) approval 2001; CE mark 2003), NeuroMatrix®, and NeuroFlex® (Stryker, FDA 510(k) approval 2014) ^10^. *In vivo* studies with NeuraGen® demonstrated equivalent functional performance in entubulation repair compared to use of standard nerve autograft and direct suture repair ^14^. NeuraGen® clinical data from several prospective and retrospective studies have been published demonstrating clinical safety and efficacy ^15– 17^. Although there appeared to be acceptable outcomes in small sensory nerve repair, mixed motor/sensory nerves fared comparatively poorly ^18^.

### Resorbable synthetic peripheral nerve guidance conduits

Synthetic biodegradable polymers offer many advantages in the fabrication of devices for peripheral nerve injury. The materials are readily available and can be engineered to modify physical and mechanical characteristics, such as strength, permeability and degradation rate as well as cell attachment and proliferation by using physical or chemical modifications ^12^.

### Polyglycolic acid

Polyglycolic acid conduits were the first to be used in clinical studies for peripheral nerve repair ^7^. There is preclinical and clinical data for Neurotube® (Synovis, CE mark 1995; FDA 510 (k) 1999) demonstrating efficacy in gap defects in animal models up to 30 mm and comparable efficacy to primary or nerve graft repair in randomised clinical trials ^19^. Although most safety and efficacy data exist for Neurotube® compared to other nerve graft conduits, the rapid degradation, concomitant reduction in mechanical strength and acidic degradation products are limiting factors to its use ^10^.

### Poly-lactic acid (PLA) and PCL

PLA and PCL are biocompatible and approved for use in numerous biomedical applications. Polylactide (L- and DL- forms) copolymerised with PCL have found utility in nerve guide conduits and Neurolac® (Polyganics, FDA 510 (k) 2003; CE mark 2004), consists of a poly (65/35(85/15 L/D) lactide ε-caprolactone) phospho-ester. Neurolac® has extensive pre-clinical *in vivo* data, and in randomised clinical trials Neurolac® is reported to have comparable efficacy to gold standard autograft in defects up to 20 mm ^20^. Known adverse events associated with the use of a Neurolac® nerve guide include but are not limited to: failure to provide adequate nerve regeneration at sites where too much tension or compression occurs; failure to provide adequate/complete nerve regeneration; transitory local irritation; infection; allergy and delayed wound healing. Limitations are reported to be the high rigidity and inflexibility of the device. In comparison to Neurolac®, Polynerve has greater tensile strength, flexibility and less acidic degradation, whilst the microgrooves on the internal lumen provide a protected environment for ingress of Schwann cells (the supportive cell of the peripheral nervous system), which align on the micro-patterned grooves and aid subsequent nerve regeneration.

### Preclinical and clinical trials

Experimental *in vitro* studies have demonstrated that PCL/PLA blended films are able to support the attachment and growth of Schwann cells ^21^. These cells are key players in nerve regeneration through delivery of neurotrophic factors and extracellular matrix proteins. Furthermore, the microgrooved internal lumen of Polynerve promotes neural regeneration ^22^ and Schwann cell alignment ^23^ to guide the nerve regeneration process. Subsequent *in vivo* studies on rat sciatic nerve gap of 10 mm demonstrate comparable efficacy of regeneration to nerve graft (current clinical gold standard) in both short (3 week) and long (16 week) timepoints ^22^.

Moreover, *in vitro* and *in vivo* evidence demonstrates an improved biological response of the specific micro-grooved architecture of Polynerve when compared to no grooves. Therefore, it is likely to be at a biological advantage when compared to the use of Neurolac® (Polyganics BV) which contains no internal lumen architecture within which to guide the neural regeneration. In randomised clinical trials, Neurolac® is reported to have comparable efficacy to gold standard autograft in defects up to 20mm ^20^. This device is made of similar polymer materials but at a different PCL:PLA ratio. Whilst Polynerve is 4:1, Neurolac® is 1:1 which confers a more rapid degradation and mechanical problems in particular a lack of flexibility. For these reasons, we expect Polynerve to be a superior nerve repair conduit in clinical use.

## Primary objective

To assess the safety and tolerability of use of the polymer biomaterial nerve conduit to repair a sensory nerve transection of the hand.

## Secondary objective

To measure efficacy of the polymer biomaterial nerve conduit to support nerve regeneration following transection of the sensory nerve of the hand.

## Protocol

### Study design

This study is a UK-based, prospective, single-centre, unblinded, phase I clinical trial of a novel nerve conduit device. The proposed study will register eligible participants to undergoing repair of a transection of a sensory nerve of the hand using a novel, synthetic nerve conduit polymer. All participants that receive the nerve conduit device will be followed for a period of 12 months post-surgery.

### Study setting

Eligible participants will be identified by the Principal Investigator and Co-Investigators within the Department of Burns, Plastics and Reconstructive Surgery at Manchester University NHS Foundation Trust (MFT) outpatient department and trauma database.

Participants deemed eligible for consideration and potential entry into the study (see Table 1) will be provided with a verbal and written explanation of the study. The participant will be given at least 4 hours and ideally greater than 24 hours to consider participation. The minimum of 4 hours is stated because these are participants with traumatic injuries and will occasionally require or be offered an operation within this time period. After all queries have been addressed and the clinical team is confident that the participant understands the study and all it’s requirements, participants will be consented onto the study.

**Table 1.** Eligibility criteria.

Inclusion criteria	Exclusion criteria
Provision of informed consent prior to any study specific procedures.	Concomitant injuries requiring surgical treatment from other specialists outwith the hand injury.
Traumatic injury/injuries to the hand with clinical suspicion of sensory nerve transection mandating surgical exploration.	Specified co-morbidities that would increase a participants risk of infection including diabetes, renal/liver disease, autoimmune diseases, primary or secondary immunocompromised participants (including immunosuppressive drugs or known disease resulting in suppressed immunity).
Male and females aged 18–80.	A stated hypersensitivity or allergy to the polymers PCL/PLA.
	Any other significant co-morbidity impacting on the risk of surgery (to be determined by the surgical team).
	Known to have participated in a clinical trial of an investigational agent or device in the previous 30 days.

## Consent

Written consent will be taken from potential participants by a member of the trial team who is suitably qualified and experienced, is good clinical practice (GCP)-trained and who has been delegated by the principal investigator to undertake this activity (this delegation will be clearly documented on the delegation log). If a participant is unable to sign the relevant consent form due to physical difficulties resulting from their clinical condition or pre-existing physical condition (e.g. visual difficulties or limb weakness), witnessed consent will be obtained.

During the consent process it will be made clear to the participant that they will remain in the trial should capacity be lost unless the decision to withdraw them is made by their representative, the research team, or by their clinical team. A model consent form, alongside the patient information sheet, is available as *Extended data* ^24^.

## Intervention

Participants recruited onto the trial will be operated on by way of routine surgical procedures in an operating theatre at MFT. Local anaesthetic (e.g. 0.5% marcaine and 2% lignocaine ± adrenaline 1:200,000) will be administered as deemed appropriate by the surgeon; and a general anaesthetic will be administered by the anaesthetic team as determined by the clinical situation.

In standard operating theatre sterile conditions, the wound will be debrided and irrigated as necessary. The digital nerve will be examined under loupe or microscope magnification and a decision made as to the most appropriate surgery method. If the nerve gap is less than 5 mm and the stumps can be co-apted in a tension-free manner, then the nerve will be repaired primarily (end-to-end). If the nerve gap is greater than 20 mm it will be repaired with a nerve graft or as has been decided between the participant and the surgeon. In the instance of a nerve gap between 5 and 20 mm, the Polynerve biomaterial nerve conduit will be used. Three diameters of Polynerve will be available sterilised and packaged: 1.5 mm, 2 mm and 3 mm diameter. All Polynerve conduits are 22 mm in length and will be cut to fit the nerve gap allowing for tension-free epineural suturing of the proximal and distal nerve ends. The nerve will be sutured into the Polynerve conduit with an 9/0 Ethilon suture (Ethicon, UK).

Skin will be repaired with standard treatment of Ethilon suture (Ethicon, UK). The wound will be covered with standard treatment of a barrier dressing such as Mepitel (Mölnlycke Health Care, Sweden), and a secure gauze-based dressing overlying. If concomitant injuries exist such as tendon injuries, then the hand will be dressed using standard treatment including post-operative splinting.

All participants will receive antibiotics at induction of anaesthetic and for 1 week post-operatively. This will be co-amoxiclav 625 mg three times daily, or if penicillin allergic clarithromycin 500 mg twice daily. This is standard treatment following nerve graft surgery.

**Figure 1.**
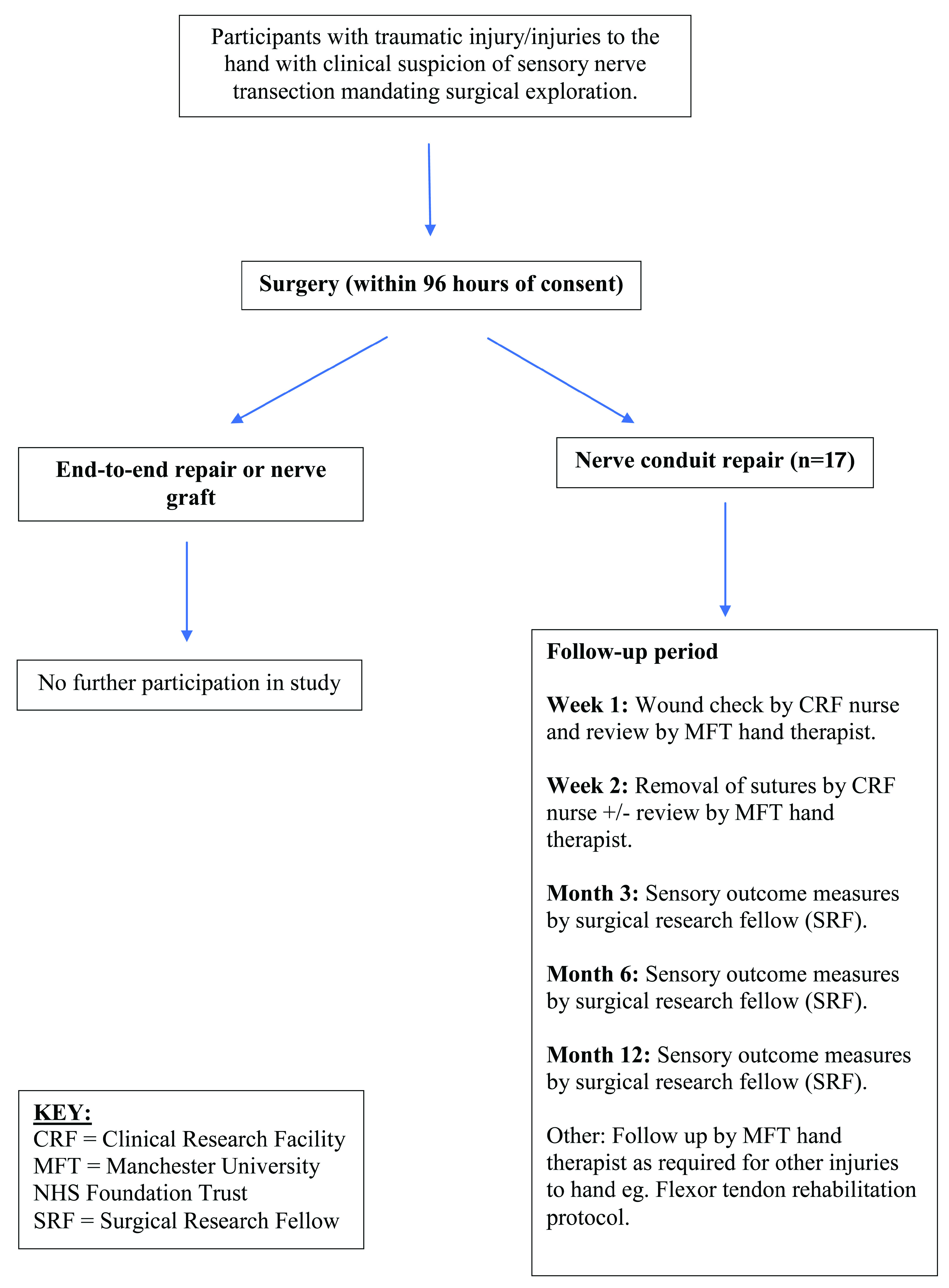
Participant timeline.

Standard post-surgical follow-up will be conducted 1 week and 2 weeks post-surgery, and additional follow up at 3 months, 6 months and 12 months post-surgery will be scheduled ( Figure 1 and Table 2).

**Table 2.** Schedule of assessments.

Assessment/Procedure	Screening / Baseline	Surgery	1 week	2 week	3 month	6 month	12 month
			± 1d	± 1d	± 7d	± 30d	± 30d
**Participant informed consent**	**X**	**X**					
**Inclusion criteria**	**X**						
**Exclusion criteria**	**X**						
**Medical and surgical history**	**X**						
**Demographics**	**X**						
**Concomitant medication**	**X**		X	X	X	**X**	**X**
**Safety reporting**	**X**		X	X	X	X	**X**
**Surgery**		**X** ^a^					
**Sensory outcome assessment**	**X**	**X** ^b^			**X**	**X**	**X**
**Ultrasound Examination**					**X**	**X**	**X**
**Wound examination**			**X**	**X**	**X**	**X**	**X**

^a^ In the event of repeat surgery, the 3-month, 6-month and 12-month follow-up visits will be performed from the date of the first surgery.

^b^ Sensory outcome assessment to be completed pre-operatively.

### Known and potential risks of study procedures

The potential risks to all participants undergoing sensory nerve repair include abnormal scar formation (hypertrophy/keloid), delayed wound healing, pain related to the wound site, neuroma formation, altered sensation of the skin around the wound (paraesthesia), no improvement in recovery of sensation, surgical site infection and bleeding requiring surgery (haematoma). Depending of the severity of these events some may require further surgery.

The additional potential risks to study participants receiving our polymer nerve conduit are persistent infection, allergy to the polymers PCL/PLA, local irritation and extrusion of the device. Some such risks may require surgical removal of the Polynerve device. These complications have not occurred in any animal studies conducted to date.

### Sample size

As this was a phase I study, primarily assessing safety of the device, a pragmatic decision on achievable recruitment numbers at a single centre who would be able to complete 12-months follow-up was made, and no formal power calculations were performed. The trial will recruit 17 participants over 12 months at an intended rate of approximately 1 to 2 participants per month. All participants fitting the eligibility criteria will be recruited.

### Data collection

***Primary outcome.*** The primary outcome is safety following implantation of the Polynerve device. Safety will be assessed based on assessment of the number and degree (based on the Clavien-Dindo classification of surgical complications ^25^) of adverse device effects (ADE) that may occur during the study period ^26^. In order to ensure all ADE are captured, all adverse events (AE) will be assessed and recorded by the P.I. or appropriate co-investigator deputized by the P.I., immediately upon notification to the study team. (A full out-of-hours AE reporting facility is made available to all trial participants following implantation of a study device.)

The investigator will assess causal relationship between the medical device under investigation and each AE.

All expected AE/ADEs of grade 3 or above, except infection of the wound any occurrences of grade 2 or above, will be recorded as a serious AE (SAE) or serious ADE (SADE). Appropriate SAE reporting procedures will be followed including sponsor, MHRA and REC reporting—this will be overseen by a Trial Management Group (TMG).

The following variables will be collected in the case report forms (CRFs) for each ADE:

- ADE diagnosis/description
- The date of ADE onset and date
- Clavien-Dindo grade of maximum intensity
- Whether the ADE is serious or not (see above)
- Assessment of relatedness to device under investigation or other procedure.
- Outcome

Safety information and all concomitant medications (that are associated with the surgical procedure) will be reviewed at each follow up visit ( Table 2).

At the end of the study data from initial treatment will be combined in the presentation of safety data. The number of participants experiencing each ADE will be summarised. The number and percentage of participants with ADEs in different categories (e.g. causally related, Clavien–Dindo ≥3 etc) will be summarised by group. SAEs/SADEs will be summarised separately if a sufficient number occur. Any ADE occurring within the defined 12-month follow-up period will be included in the ADE summaries.

***Secondary outcome.*** To measure degree of efficacy of the nerve conduit device, standard sensory outcome measures will be used:

- two-point discrimination (2PD) ^27^,
- the Weinstein Enhanced Sensory Test (WEST) ^28^,
- Locognosia test (adapted methodology from Jerosch-Herold *et al.* JBJSB 2006)

These measurements will be assessed at baseline/time of surgery and at 3-, 6- and 12-months after implantation of the device ( Table 2). Data will be tabulated and appropriate statistical analysis performed.

## Demographic data

Characteristics of the participants, including medical history and disease characteristics at baseline will be listed for each participant and summarised for the whole cohort.

### Data management

A paper-based data capture system will be used for data collection and query handling.

Completed CRFs submitted to the clinical trials unit will be entered onto a study database by the trial data manager. The study database is stored on a secure server with controlled access, regular back-up of data and an audit trail of changes made to the data.

### Statistical methods

Demographic data will be tabulated. All primary outcome data will be described and summarised. Secondary outcome measurement data will be tabulated and mean ± SD of static 2PD, WEST and Locognosia compared to contralateral control nerves will be presented with one-way ANOVA analysis between groups.

### Data and safety monitoring

Trial participants will have 24-hour access to a plastic surgery registrar and consultant at Manchester University NHS Foundation Trust in case of any AE/ADE. Any treatment or further surgery required will be decided by the C.I. and implemented at the earliest available opportunity. Appropriate safety reporting will be followed by this on-call team.

### Oversight committees

***TMG.*** A TMG will be established and will include those individuals responsible for the day-to-day management of the trial (including the chief investigator, co-investigators, the trial statistician and the study project manager). The TMG have operational responsibility for the conduct of the trial including monitoring overall progress to ensure the protocol is adhered to and to take appropriate action to safeguard the participants and the quality of the trial.

***Independent data monitoring committee (IDMC).*** An IDMC will be instituted to review accruing trial data and to assess whether there are any safety issues that should be brought to the participants’ attention, whether any safety amendments should be made or if there are any reasons for the trial should not continue. The IDMC will be independent of the investigators, funder and sponsor.

### Harms

Expected AEs which may occur as a result of the surgical procedure include:

•   Bleeding from the wound site (haematoma)

•   Infection of the wound site

•   Abnormal scar formation (hypertrophy/keloid)

•   Delayed wound healing

•   Pain related to the wound site

•   Altered sensation of the skin around the surgical site (paraesthesia)

•   Development of neuroma

Expected ADEs which may occur as a result of the medical device under investigation are:

•   Intractable infection of the surgical site

•   Allergy to the polymers PCL/PLA

•   Local irritation

•   Extrusion of device through the wound

### Auditing

Authorized representatives of the sponsor, regulatory authority, or an ethics committee may perform audits or inspections at the centre, including source data verification.

## Ethics and dissemination

The trial will be conducted in accordance with the principles of GCP.

The South Manchester REC has approved the trial protocol, informed consent forms and other relevant documents e.g. advertisements and GP information letters (REC reference: 17/NW/0111). In addition, the Health Research Authority (HRA) has also given approval for the trial to commence recruitment.

### Regulatory compliance

Clinical trial authorisation (CTA) has been obtained from the Medicine and Healthcare products Regulatory Agency (MHRA). The protocol and trial conduct will comply with the Medicines for Human Use (Clinical Trials) Regulations 2004 and any relevant amendments.

### Protocol amendments

Any changes in research activity will be reviewed and approved by the Chief Investigator. With the oversight of the sponsor, the subsequent amendment will be categorised as substantial or non-substantial. Any required changes to the CTA or the documents that supported the original application for the CTA and/or ethical approval will be submitted as an amendment to the appropriate ethical and regulatory authorities by the clinical trials unit. Substantial amendments will not be implemented until the REC grants a favourable opinion for the study, confirmation of No Objection is received from MHRA and local R&D department approval.

### Confidentiality

Participants will be assigned a unique trial ID via the King's College clinical trials unit randomisation service which will be used throughout their participation in the trial. Any personal data recorded will be regarded as confidential, and any information which would allow individual participants to be identified will not be released into the public domain.

### Access to data

Essential documents are documents that individually and collectively permit evaluation of the conduct of the trial and substantiate the quality of the data collected. Essential documents will be maintained at the Manchester Clinical Trials Unit (CTU), University of Manchester and at the investigator site in a way that will facilitate the management of the trial, audit and inspection. These documents must be retained for a sufficient period of time (at least 15 years) for possible audit or inspection. Documents will be securely stored and access restricted to authorised personnel.

### Dissemination policy

The main study results will be published in the name of the study, in a peer-reviewed journal, on behalf of all collaborators. The manuscript will be prepared by a writing group, appointed from amongst the TMG.

## Discussion

**Figure 2.**
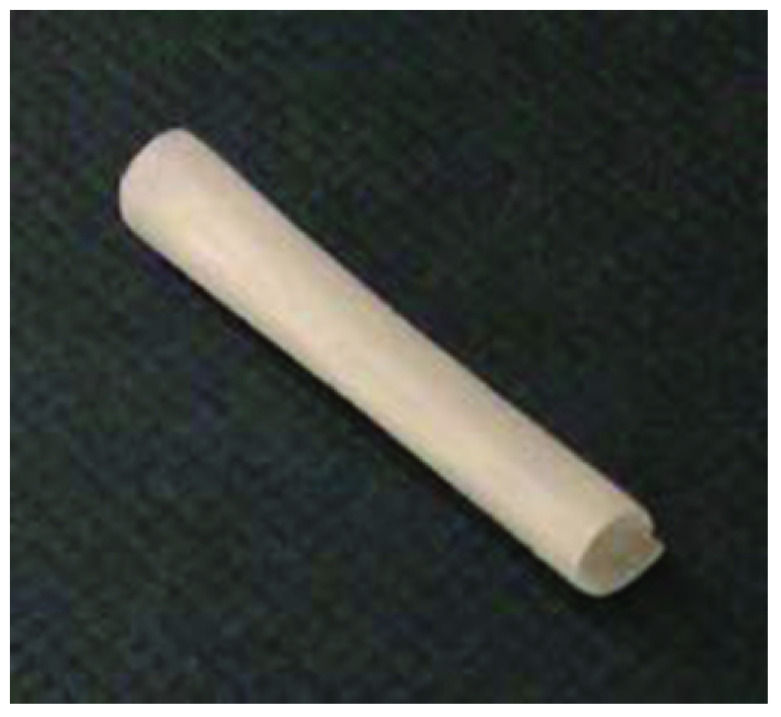
Image of Polynerve. This image demonstrates a standard Polynerve device of 2 mm diameter and 22 mm long (image not to scale).

The proposed intended use of Polynerve is to surgically repair nerve gaps of up to 20 mm. Nerves selected for the Phase 1 clinical trial of this device will be small sensory nerves of the hand. Polynerve ( Figure 2) is designed to support the nerve regeneration process by providing a protected environment for ingress of Schwann cells (the supportive cell of the peripheral nervous system), which align on the micro-patterned grooves and support subsequent nerve regeneration. It is designed to be absorbed by the body completely 18 months after implantation by which time nerve regeneration is complete.

Development of an improved-quality, faster peripheral nerve repair using a specialised biodegradable nerve conduit has the potential to transform outcomes of peripheral nerve injury.

Our aims are to improve regeneration across a nerve gap, reduce participant morbidity through avoiding the need for a donor nerve grafting operation thereby avoiding donor site scarring and loss of donor nerve function. Of additional benefit will be an improved surgical efficiency in the operating theatre, because the additional nerve graft harvest procedure will not be necessary.

## Data availability

### Underlying data

No underlying data are associated with this article.

### Extended data

Open Science Framework: A phase I trial of a novel synthetic polymer nerve conduit ‘Polynerve’ in participants with sensory digital nerve injury (UMANC). https://doi.org/10.17605/OSF.IO/HG6WB ^24^.

This project contains the following extended data:

- UMANC Informed Consent Form V5 0 13-Aug-2018.doc
- UMANC patient information V5 0 13-Aug-2018.doc

### Reporting guidelines checklist

Open Science Framework: SPIRIT checklist for Phase I trial of a novel synthetic polymer nerve conduit ‘Polynerve’ in participants with sensory digital nerve injury (UMANC): Study protocol. https://doi.org/10.17605/OSF.IO/HG6WB ^24^.
