# Supplementary material for: Protocol for a phase I trial of a novel synthetic polymer nerve conduit 'Polynerve' in participants with sensory digital nerve injury (UMANC)
Source: F1000Res. 2019 Jun 24;8:959. [Version 1] doi: 10.12688/f1000research.19497.1 (PMC7355221; doi:10.12688/f1000research.19497.1)
Supplement: Peer review history

# Peer review: Reviewer response for version 1

**DOI:** 10.5256/f1000research.21376.r50341

**Published:** 2020-07-10

**Authors:** Andrew M. Hart

**Affiliations:** 1 The Centre for Cell Engineering, College of Medical, Veterinary and Life Sciences, University of Glasgow, Glasgow, UK; 2 NHS Greater Glasgow & Clyde, Glasgow, UK; 3 The Scottish National Brachial Plexus Injury Service, Glasgow, UK

- This is a sensible safe approach to the phased development of a novel nerve reconstruction device. It stands in contrast to other products that have been introduced without due testing.
- Functional outcome measurement is challenging, the existing tests being insufficiently specific for fine discriminatory purposes, but the authors suggest a sensible pragmatic approach for this phase of study.
- Safety is well addressed.
- Ethics are well addressed.
- Data governance is addressed.

Is the study design appropriate for the research question?

Yes

Is the rationale for, and objectives of, the study clearly described?

Yes

Are sufficient details of the methods provided to allow replication by others?

Yes

Are the datasets clearly presented in a useable and accessible format?

Yes

Reviewer Expertise:

I am a peripheral nerve surgeon in a national service, with 20 years experience of neurobiological research, focused around nerve injury and tissue engineering.  I edit the world's second largest reconstructive Journal.

I confirm that I have read this submission and believe that I have an appropriate level of expertise to confirm that it is of an acceptable scientific standard.

# Peer review: Reviewer response for version 1

**DOI:** 10.5256/f1000research.21376.r50340

**Published:** 2020-03-20

**Authors:** Daniel F. Kalbermatten

**Affiliations:** 1 Department of Plastic, Reconstructive, Aesthetic, and Hand Surgery, Basel University Hospital, University of Basel, Basel, Switzerland

This manuscript authored by Murphy *et al.* is a study protocol for a first-in-human use of a new device called "Polynerve“ for nerve gap bridging. 

 Its primary objective is to describe a clinical trial to evaluate the safety and tolerability of use of a new polymer biomaterial nerve conduit to repair a sensory digital nerve gap of the hand. Secodarily, the trial aims at measuring the efficacy of this innovative conduit as an alternative to surgical repair, autologous grafting and other allogenic materials to bridge small gaps of digital nerves. 

 Overall, the study design is well-structured and the rational of utilizing this new method in a clinical pilot study is clearly explained and outlined.

 The introduction gives an overview of important impact socio-economic of nerve injury for the affected individual and the societal interest of finding alternative methods to restore continuity in nerve defects and minmize functional and productive loss. Here, reference 3 is quoting a study on the impact of median and ulnar nerve lesions in the forearm, a very severe subset of injury which may yet not be representative for the majority of over 9000 nerve lesions occuring in the UK annually.

 The authors provide a detailed overview of the requirements of a valuable alternative  method avoiding the disadvantages of current treatment methods, such as autologous grafting or commonly used tubulization devices, including non-resorbable and resorbable nerve conduits. In addition, the authors may also mention the modern options of nerve allografting as an emerging method, especially for smaller sized gaps of sensory nerves, e.g. as recently described by Mauch JT *et al.* (see reference 1 ^1^). 

 Nevertheless, the discussion clearly explains the rational for using the new material in the planned trial by the specific advantages of Polynerve as a blend of poly-captrolactone (PCL) and poly-lactic-acid (PLA) promoting neural regeneration and Schwann cell alignment  to guide the process of axon regeneration by lumen consisting of a special micro-grooved architecture. 

 The further paragraphs about the exact design and setting of the study including consent and intervention with a detailed participant timeline and known risks of the procedures are thorough and concise. The methodology regarding data collection and management and statistics are very solid, as well as the description of oversight committees and dissemination.

 The protocol is completed by a short discussion which summarizes the expected superior  qualities of this new biopolymer nerve conduit and the main goals of a promising future study on nerve regeneration.

Is the study design appropriate for the research question?

Yes

Is the rationale for, and objectives of, the study clearly described?

Yes

Are sufficient details of the methods provided to allow replication by others?

Yes

Are the datasets clearly presented in a useable and accessible format?

Not applicable

Reviewer Expertise:

Experimental and clinical trials in peripheral nerve repair and regeneration, bioarbsorbable nerve conduits in nerve gap repair

I confirm that I have read this submission and believe that I have an appropriate level of expertise to confirm that it is of an acceptable scientific standard.

## References

[1] : A Systematic Review of Sensory Outcomes of Digital Nerve Gap Reconstruction With Autograft, Allograft, and Conduit. *Ann Plast Surg*.82(4S Suppl 3) : 10.1097/SAP.0000000000001851 S247-S255 10.1097/SAP.0000000000001851 30855395

# Peer review: Reviewer response for version 1

**DOI:** 10.5256/f1000research.21376.r56466

**Published:** 2019-12-11

**Authors:** Dominic M. Power

**Affiliations:** 1 The Birmingham Peripheral Nerve Injury Service, Queen Elizabeth Hospital Birmingham, Birmingham, UK

The protocol is well-written and explains the trial design and the rationale for the study well.

 In the introduction the statement that Ref 3 costs of care are estimated at 51K euros needs clarification. This is the cost for a median nerve injury and includes societal costs and loss of production. I think following on from the statement regarding 9000 injuries per annum that this is misleading and requires clarification.

 The paragraph detailing the option of direct repair should be expanded to discuss the challenges in direct repair of acute nerve injuries. Delay to repair renders the tension greater and the need for debridement may result in unacceptable tension. There are strategies that can be employed to reduce repair site tension and these include using conduits or connectors to bridge the repair site with offloading remote sutures. Papers by Zhu ^1^ and by Neubrech ^2^ detail the efficacy of this approach.

 "Where the nerve gap exceeds more than 5 mm, there are two fundamental options, either ‘nerve grafting’ or ‘tubulisation’" I am concerned that this statement may mislead the reader. A gap of 5-6mm may be left in a no gap acute nerve injury with entubulation as a way of detensioning a repair site. The use of conduits to allow bridging of gaps up to 6mm is supported by evidence form Weber and others. Longer gap entubulation is less favourable and beyond 12mm the evidence for existing conduits is poor. The surgeon should consider the options and these are well described in the paper by Ducic *et al.* ^3^ They include using autograft and also allograft for sensory nerves (limited evidence for mixed nerves).

 I feel that the introduction is too superficial and aims to prepare the reader for a discussion of an improved conduit design but at the cost of an inadequate review of the available options and evidence base. At minimum there should be an analysis of the gap sizes in the Weber paper (poor data interpretation and grouping of larger and medium gaps with small numbers). A recent systematic review on conduits and gap management by DeSlivia *et al., *2015 ^4^ is better to discuss. Particularly as there is recruitment of the 5-20mm gap range a more extensive introductory comment and literature review is warranted.

 The discussion on the non-absorbable versus bioresorbable conduits is comprehensive.

 There should be commentary on the sizing of conduits and the issues about transparency assisting use, particularly when there is a use of a conduit in a short gap reconstruction where misalignment can cause problems.

 There is good explanation of the rationale for the PCL use.

 There should be clarification of the need for a conduit to allow minimal deformation under loading to prevent disruption of the primitive fibrin clot matrix for axon regeneration. The statement about the mechanical properties of the Polynerve is insufficient and should include a discussion of this aspect of load and deformation.

 Intervention:

 There is no discussion of nerve debridement.

 There is no discussion of the number and technique of suture placement to secure the conduit, nor needle type to pass through the conduit. As such the study may not be repeatable by another group.

 Is the antibiotic use for 1 week local policy or is this adapted for this patient population given the use of a synthetic conduit.

 Risks:

 There is comment on the risk of neuroma in sensory nerve repair. The specific risks of the Polynerve shouldn't include the general risks associated with a bioresorbable conduit as listed but further detail of the risk of nerve displacement from the conduit should be detailed. Extrusion typically refers to ulceration of the conduit through the wound and not to the nerve displacement from the conduit.

 There is no discussion of the type of US, the technique and findings interpretation as a secondary outcome measure other than listing in the schedule of events. This is not a normal investigation following simple nerve repair or grafting and the rationale and interpretation must be discussed here.

 The remainder of the monitoring TMG, DMEC, audit and AE reporting is excellent.

 The conclusion is good but the points raised should be introduced earlier for the reader to understand the concept for this conduit. The failure to review the evidence for allograft is an oversight.

Is the study design appropriate for the research question?

Yes

Is the rationale for, and objectives of, the study clearly described?

Yes

Are sufficient details of the methods provided to allow replication by others?

Yes

Are the datasets clearly presented in a useable and accessible format?

Not applicable

Reviewer Expertise:

Clinical trials in peripheral nerve repair and regeneration, conduits and allograft in nerve gap repairDetensioning nerve reapir, sutureless nerve repair

I confirm that I have read this submission and believe that I have an appropriate level of expertise to confirm that it is of an acceptable scientific standard, however I have significant reservations, as outlined above.

## References

[1] : Nerve wrap after end-to-end and tension-free neurorrhaphy attenuates neuropathic pain: A prospective study based on cohorts of digit replantation. *Scientific Reports*.2018;8(1) : 10.1038/s41598-017-19134-8 10.1038/s41598-017-19134-8 29330413PMC5766600

[2] : Enhancing the Outcome of Traumatic Sensory Nerve Lesions of the Hand by Additional Use of a Chitosan Nerve Tube in Primary Nerve Repair: A Randomized Controlled Bicentric Trial. *Plast Reconstr Surg*.142(2) : 10.1097/PRS.0000000000004574 415-424 10.1097/PRS.0000000000004574 30045179

[3] : Refinements of nerve repair with connector-assisted coaptation. *Microsurgery*.2017;37(3) : 10.1002/micr.30151 256-263 10.1002/micr.30151 28035702

[4] : Decellularized Nerves for Upper Limb Nerve Reconstruction: A Systematic Review of Functional Outcomes. *J Reconstr Microsurg*.2015;31(9) : 10.1055/s-0035-1558463 660-7 10.1055/s-0035-1558463 26280520
